# Book Notices

**Published:** 1892-08

**Authors:** 


### Book Notices.

THE ANNUAL OF THE UNIVERSAL MEDICAL SCIENCE, issued every year by Dr. Chas. E. Sajous, Philadelphia, and published by F. A. Davis Company, has been received for 1891, and will have attention in an early number of this JOURNAL. We note that Dr. Sajous has taken quarters in Paris and can be addressed in the care of Drexel, Harjes & Co., 31 Boulevard Hausmann. His object in visiting Paris—where he will remain—and in other parts of Europe several years, is to arrange to bring out the Annual, in future, in French as well as in English. Dr. Sajous writes us:

"The contributions of American physicians do not obtain the recognition to which they are fairly entitled on the Continent. This is not due to wilful disregard of the work done in this country, but mainly to the fact that the English language is not read as easily as French or German. The plan of the Annual naturally brings within a limited scope a great number of American writers, but few of whom are quoted abroad, and I hope, by presenting an edition of the work in the French language, soon to obtain for our confreres the recognition due them.

"A feature of importance in the next Annual will be the insertion, when possible, of the address of each author quoted. You can readily appreciate the value of this new step. John Smith, of Lebanon, Pa., can be distinguished from hundreds of the same name practicing in this country and England. The individual is properly recognized—so is his country. Were each journal to give the address of its contributors, this improvement could easily be effected."

DANIEL'S TEXAS MEDICAL JOURNAL always gives the P. O. address of its contributors.

---

KEATING'S NEW PRONOUNCING DICTIONARY OF MEDICINE has just made its appearance from the press of W. B. Saunders, of Philadelphia. Its advent has been long looked for and will be hailed with delight. In view of the great advances in medicine, and especially in the department of physiology, bacteriology and microscopy, and the introduction of new words, the dictionaries in use are out of date and almost worthless. Keating's is up with the front and will be found to contain many words recently come into use, and fuller definitions of usual medical terms. The book is printed in clear readable type, which is a matter of prime importance yet not always sufficiently considered by publishers. In the appendix, which is very full and complete, will be found much useful matter not generally found in such works. The price is, cloth $5; leather, $6 net. We predict for this work a large demand and ready sale. Every physician ought to have a copy. Keating's other work, "Diseases of Children," took so well and gave such satisfaction, his name alone will sell this new dictionary.

